# 11β-hydroxysteroid dehydrogenase type-II activity is affected by grapefruit juice and intense muscular work

**DOI:** 10.1590/2359-3997000000296

**Published:** 2017-09-04

**Authors:** Christopher Kargl, Mohammad Arshad, Fahad Salman, Regina C. Schurman, Pedro Del Corral

**Affiliations:** 1 Department of Biological Sciences Benedictine University Lisle IL Department of Biological Sciences, Benedictine University, Lisle, IL

**Keywords:** Cortisol, cortisone, exertion, stress, citrus flavonoids

## Abstract

**Objective:**

The enzymatic activity of 11β-hydroxysteroid dehydrogenase-2 (11β-HSD2) is key to protecting mineral corticoid receptors from cortisol and has been implicated in blood pressure regulation. Grapefruit juice (GFJ) and acidity are thought to inhibit this enzyme *in vitro*. This study examines the effect of GFJ and intense exercise on 11β-HSD2 enzyme activity *in vivo*.

**Subjects and methods:**

Eighteen subjects ingested GFJ or apple juice (CON) on separate days prior to reporting to the laboratory in a randomized order. Saliva (Sal) samples were obtained at baseline, 15 and 45 minutes post-treadmill stress test; Sal cortisone (E) and cortisol (F) levels were determined, and the Sal cortisone:cortisol (E:F) ratio was used as an index of 11β-HSD2 enzyme activity at rest and after intense muscular work.

**Results:**

GFJ treatment decreased baseline 11β-HSD2 enzyme activity (44%) and Sal-E (28%) compared to CON (both, *p* < 0.05). Sal-E (r = 0.61, *p* < 0.05) and Sal-F (r = 0.66, *p* < 0.05) were correlated with diastolic blood pressure (DBP) in GFJ-treated individuals. Treadmill stress significantly increased Sal-E and Sal-F but did not alter 11β-HSD2 enzyme activity regardless of treatment. When treatments were examined separately, CON 11β-HSD2 enzyme activity decreased by 36% (*p* < 0.05) from baseline to 15 post-treadmill exercise.

**Conclusion:**

Our findings suggest that GFJ and intense muscular work decrease 11β-HSD-2 activity independently, and no additive effect was noted. The association between DBP and the levels of Sal-F and Sal-E during the GFJ trial should be interpreted cautiously and warrants further investigation.

## INTRODUCTION

The enzyme 11β-hydroxysteroid dehydrogenase 2 (11β-HSD2) is expressed in mineralocorticoid target tissues such as the kidney, colon, salivary glands, and placenta. This enzyme oxidizes cortisol (F) into inactive cortisone (E), thereby rendering the mineralocorticoid receptor-hormone complex inactive (1). The enzymatic activity of 11β-HSD2 can be estimated using the ratios of these hormones in urine (2-5) and saliva (Sal) fluids (4,6,7). Subtle deficiencies in 11β-HSD2 activity have been reported in subsets of hypertensives and normotensives (8,9), whereas more severe hypertension and hypokalemia are observed in cases of substantial or complete loss of 11β-HSD2 activity (1,9).

Preliminary *in vitro* (10) and *in vivo* (5,10) studies suggest that grapefruit juice (GFJ) transiently decreases 11β-HSD2 enzyme activity, and this has been associated with high levels of bioflavonoids, such as naringin and its aglycone, naringenin. *In vivo* pilot studies have been limited by small sample sizes of 1-6 research subjects and have used large (1-2 L/day) amounts of GFJ (5,10). A study using a larger sample size and a moderate (0.7 L/day) GFJ intake along with a more convenient matrix, such as Sal sampling, to assess 11β-HSD2 activity is warranted.

It has been reported that acidosis decreases 11β-HSD2 activity in the human placenta and in rodent inner medullary-collecting duct cells (11,12). Intense muscular work is associated with transient perturbations in the acid-base balance, in which the pH can drop below 7.0, and is related to lactic acidemia (≥ 15 mmol/L) (13). It is conceivable that intense muscular work may decrease 11β-HSD2 enzyme activity. Taken together, little is known about the regulation of 11β-HSD2 enzyme activity *in vivo* in humans. Given the importance of this enzyme on blood pressure regulation in adult (8,9) and pediatric (3) populations, further studies are warranted. This study had the following aims: 1) to examine the effect of moderate GFJ ingestion compared to a control treatment of apple juice (CON) on sal 11β-HSD2 enzyme activity in a group of healthy volunteers under resting conditions; and 2) to examine the effect of intense muscular work, which is known to induce lactic acidemia in response to 11β-HSD2 enzyme activity in tests using GFJ vs CON treatments. Based on a preliminary analysis of the literature, we hypothesized that GFJ intake will be decreased more by sal 11β-HSD2 activity than by the CON treatment and that intense muscular work will inhibit 11β-HSD2 activity to a greater extent after GFJ intake than in the CON.

## SUBJECTS AND METHODS

### Subjects

Eighteen research volunteers were recruited to participate in this study. The subject characteristics are presented in Table 1. Informed consent was obtained and approved by the Institutional Review Board of Benedictine University in accordance with the international code of ethics (Declaration of Helsinki). Criteria for participation included ages between 18 and 55, BMI between 18 and 30, non-smokers, no hypertension/diabetes, and no use of oral or topical glucocorticoids for the last 3 months. Exclusion criteria included metabolic, endocrine, renal, cardiopulmonary, or orthopedic diseases that prevent intense exercise. Subjects taking medication or dietary supplements known to alter F metabolism or taking drugs that could be altered by GFJ were disqualified from study participation.

**Table 1:** Subjects physical and cardiovascular characteristics

Number of subjects		18 (15 males & 3 females)	
Age (years)		31.1 ± 2.7	
BMI		24.5 ± 0.9	
% body fat		18.5 ± 1.8	
**Treatments**			

**Grapefruit Juice (GFJ)**		**Apple (CON)**	

Systolic blood pressure (mmHg)	124 ± 2		124 ± 2
Diastolic blood pressure (mmHg)	79 ± 2		78 ± 1
Mean arterial pressure (mmHg)	94 ± 2		94 ± 2
Heart rate (beats/min)	63 ± 3		65 ± 3

### Study design

**Figure 1:**
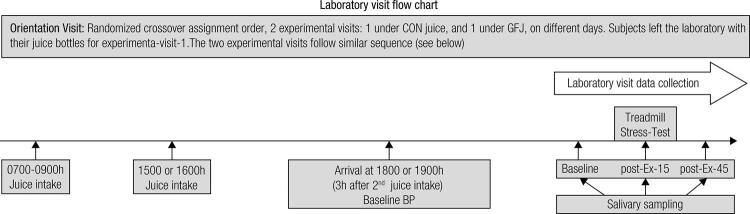
Experimental design flow-chart for visits 1 and 2. Grapefruit juice (GFJ). Each GFJ bottle contained 375 mL of 100% Pink Pure Grapefruit Juice, 1 ingested after breakfast, and 1 ingested mid-afternoon, 3h before their scheduled visit. For CON, each bottle contained 300 mL of 100% apple juice from concentrate, see Study Design, under Material & Methods for additional information.

This study used an open label, randomized, crossover assignment (ClinicalTrials.gov identifier: NCT02187328). Each subject reported to the laboratory on three separate days. The first visit was an orientation visit in which the subjects were walked through the protocol step-by-step. There were two experimental visits (Figure 1) that were preceded by a dietary intervention. In one of the experimental visits, subjects ingested GFJ (375 mL of Pink Pure Grapefruit Juice; Lakewood Farms, Miami, FL) after breakfast (0700 h – 0900 h), at mid-afternoon, and 3h before their scheduled visit. The GFJ was made from organic grapefruits that were fresh-pressed (not from concentrate) and unsweetened. In the other experimental visit (5 to 20 days apart), subjects performed the CON intervention (300 mL of 100% Apple juice from concentrate; Wal-Mart Stores, Inc., Bentonville, AR) under the same experimental conditions to equalize the carbohydrate content between the trials but keep differences in naringin content. Subjects reported to the laboratory at 1800 h or 1900 h (~5-5.5 h after lunch). Upon arrival, the subjects rested quietly for 5 minutes prior to obtaining supine blood pressure (cuff size 17-42 cm) and heart rate measurements using a validated oscillometric automated device (Omron BP791IT; OMRON Healthcare, INC. Lake Forest, IL 60045). Two treadmill stress tests were conducted to determine the maximal oxygen uptake (VO_2_ max). The subject breathed through a non-rebreathing valve while wearing a facemask so that expired gases were analyzed continuously by previously calibrated O_2_ and CO_2_ analyzers (TrueOne 2400; ParvoMedics, Sandy, UT). Subjects jogged/ran at 7.2-12.0 km^.^h^-1^ with a 0% grade (depending on fitness level) for 3 min, and the treadmill speed was subsequently increased by 1.6 km^.^h^-1^ for the next two 3 min stages. Thereafter, treadmill speed was held constant and the grade was increased by 3% every 3 minutes until volitional exhaustion was achieved, after which treadmill speed and grade were reduced to 4.8 km^.^h^-1^ and 0% for 1 minute before the test was ended and a 10 µL blood sample was collected to measure blood lactate levels (Lactate Plus analyzer; Nova Biomedical, Waltham, MA).

### Sample collection and analysis

For each experimental visit, samples were collected at baseline and 15 min (Post-15) and 45 min (Post-45) post-treadmill stress test. Subjects drank 100-200 mL of water at least 10 min prior to collection of the baseline sample, and immediately after, the Post-15 sample was collected. Sal samples were collected using a Salivette device (Sarstedt, Newton, NC) as described by the manufacturer, and then centrifuged and stored at -20 °C until analyzed. Sal-F and Sal-E were analyzed using an enzyme immunoassay and chemiluminescence with commercially available kits (Arbor Assays, Ann Arbor, MI). Both assays used 50 µL samples, and the assays were performed in duplicate per the manufacturer’s instructions. The manufacturer reported that F antibody cross-reactivity for E was 1.2% and the sensitivity of the assay was 0.0477 nmol/L. For the E antibody, cross reactivity for F and corticosterone was < 0.1%, and the sensitivity of the assay was 0.0293 nmol/L. The intra-assay coefficients of variation were 5.6% (F) and 5.9% (E). The enzymatic activity of 11β-HSD2 was calculated as the ratio of E/F (2-6). Tests for equality of variances were run, and ANOVA and paired t-tests were used when appropriate to examine differences in E, F, and 11β-HSD2 enzyme activity. Paired t-tests were conducted to test the basic vital statistics between trials, and Pearson correlations were used to examine relationships between saliva hormones (and 11β-HSD2 enzyme activity) and blood pressure parameters. Statistical significance was set at α = 0.05. The results are presented as means ± standard error (SE).

## RESULTS

### Blood pressure and heart rate

There were no significant differences in resting blood pressure and heart rate between the GFJ and CON treatment groups (Table 1).

### Stress test

Subject VO_2_max (48.1 ± 2.1; 48.9 ± 2.1 ml^.^kg^.^min^-1^), maximal heart rate (185 ± 3; 187 ± 3 beats^.^min^-1^), and post-exercise lactate levels (10.8 ± 0.8, 11.5 ± 0.4 mmol/L) were similar between the CON and GFJ trials, respectively.

### Saliva glucocorticoids

**Figure 2:**
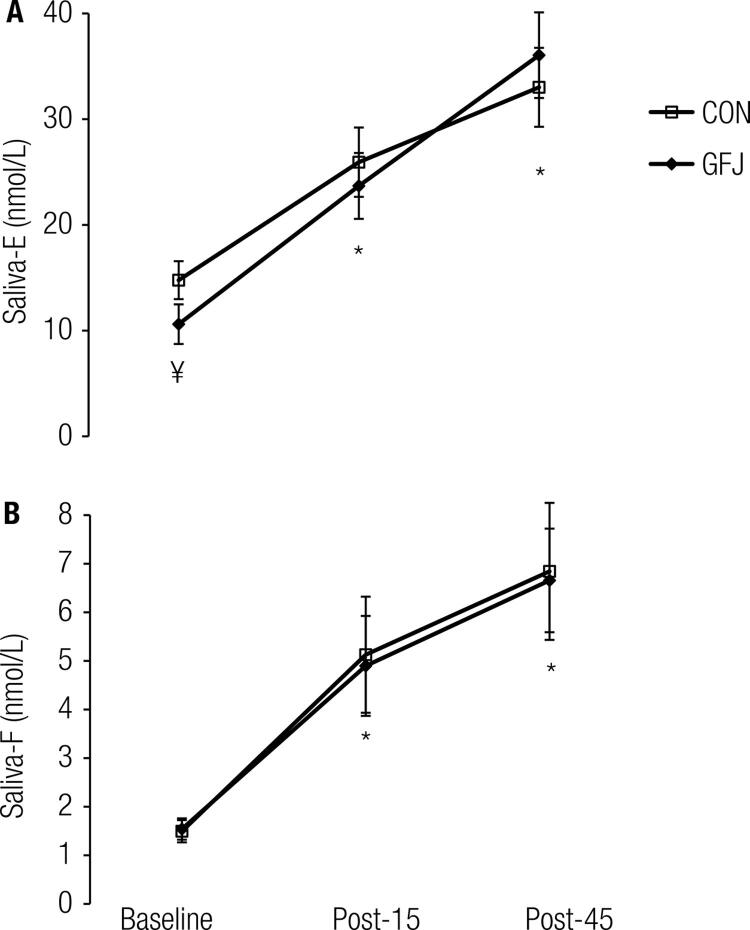
(A) Saliva-E; (B) Saliva-F at baseline, 15 min post and 45 min post treadmill stress test. Close diamonds GFJ; Open squares CON. * Denotes significant difference (*p* < 0.05) compare to baseline; ¥ denotes significant difference (*p* < 0.05) between GFJ and CON at baseline.

**Figure 3:**
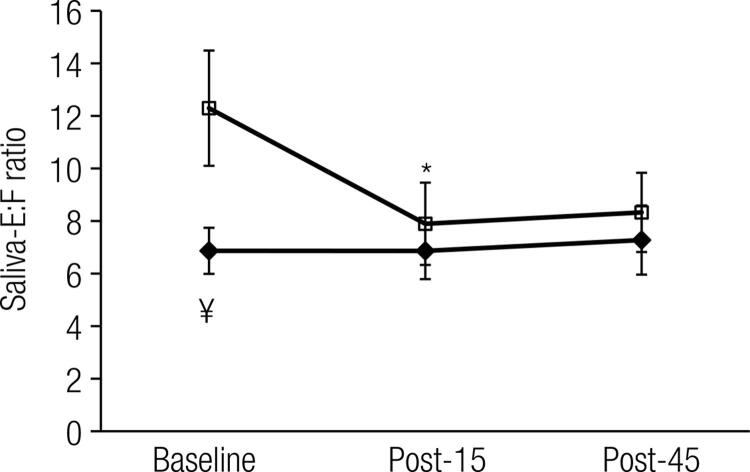
Saliva E:F ratio at baseline and at 15 min post- and 45 min post-treadmill stress test. Closed diamonds = GFJ; Open squares = CON. ¥ denotes significant differences (*p* < 0.05) between GFJ and CON at baseline; * denotes significant differences (*p* < 0.05) compared to baseline for the CON treatment.

Sal-E increased over time (*p* < 0.05) from baseline in GFJ and CON (Figure 2A). Similarly, the Sal-F concentration increased over time from baseline (*p* < 0.05) in the GFJ and CON trials (Figure 2B). For both hormones, within each trial, the results at the Post-15 and Post-45 time points differed from their corresponding baselines. However, no differences were found between the GFJ and CON trials (*p* > 0.05). When the isolated effect of treatment on the baseline hormone concentration was examined, a significant difference was noted for Sal-E (*p* < 0.05; one-tail, paired t-test). Figure 3 shows the E:F ratio at baseline and during the post-treadmill stress test. Overall, ANOVA indicated that there was no statistically significant difference between GFJ and CON. However, when we examined the independent effect of treatment on the baseline E:F ratio, a statistically significant difference was found (*p* < 0.05), suggesting that GFJ inhibited baseline 11β-HSD2 enzyme activity. Similarly, when we examined the effect of intense muscular work during the CON trial, we found that the E:F ratio decreased from baseline to Post-15 (*p* < 0.05).

### Correlations

**Figure 4:**
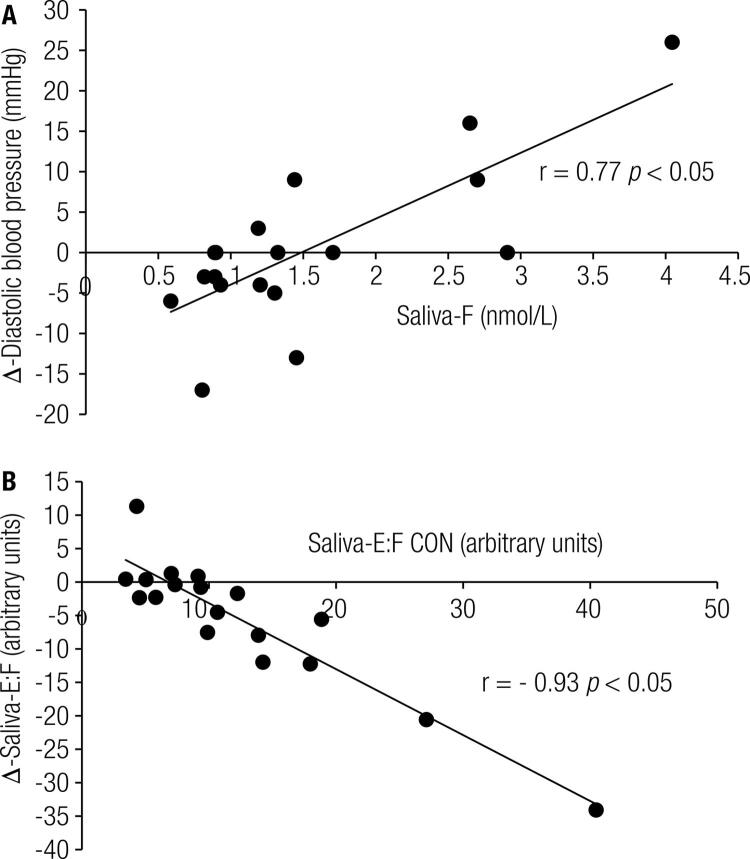
(A) Correlation between Saliva-F and the ∆-DBP between trials, r = 0.77, *p* < 0.05; (B) Correlation between baseline Saliva-E:F ratio during CON and ∆-Sal-E:F ratio between CON and GFJ (r = - 0.93, *p* < 0.05).

Sal-E (r = 0.613, *p* = 0.007) and Sal-F (r = 0.658, *p* = 0.003) were correlated with diastolic blood pressure (DBP) in GFJ but not in CON. When CON and GFJ samples were pooled (n = 36), Sal-E (r = 0.337, *p* = 0.045) and Sal-F (r = 0.333, *p* = 0.047) were correlated with DBP. Figure 4A shows that there was a significant correlation between Sal-F during the GFJ trial and the ∆-DBP between trials. Figure 4B shows a significant inverse correlation between the E:F ratio at baseline during CON and the ∆- for the E:F ratios (Sal E:F ratio CON - Sal E:F ratio GFJ).

## DISCUSSION

Our baseline CON values for the E:F ratio were similar to those previously reported by others (6). The short term GFJ treatment in the present study lowered enzyme activity by 44% (Figure 3), which compares favorably to the ~40% inhibition estimated from the urinary-E/F ratio obtained in a pilot study (10) and to the ~60% obtained in a patient presenting with edema and hypokalemia associated with the habitual oral intake of 1 liter/day of GFJ (5). Taken together, our findings suggest that Sal effluents are a cost-effective and convenient matrix (compared to plasma/urine) for probing alterations in 11β-HSD2 activity induced by GFJ ingestion. In our study, the decrease in enzyme activity induced by GFJ at baseline was mainly driven by a significant decrease (~30%) in Sal-E concentration. This is in line with a decrease in F oxidation to E, a reaction that is mediated by 11β-HSD2 (1,9,10) and likely inhibited by one or more flavonoids found in GFJ (naringenin, quercetin, hesperetin, and apigenin), all of which have been shown to inhibit 11β-HSD2 activity *in vitro* (10). Urine, Sal-F, and Sal-E are determined by plasma-free F & E fractions and 11β-HSD2 activity. The plasma-free F & E fractions are in turn determined by CBG. Given the short time (10 h) between the morning GFJ ingestion and Sal sample collection and the half-life of CBG (1-3 days), it is unlikely that the effects of GFJ on Sal-E and Sal-F are linked to changes in CBG concentration. Finally, GFJ is well known for its inhibitory effects on intestinal CYP3A4 enzyme activity, which leads to numerous drug interactions (14), and this enzyme is also involved in the metabolism of E and F to 6β-hydroxy-E and 6β-hydroxy-F, respectively. It has also been shown that GFJ decreases the urinary ratio of 6β-hydroxy-F to F in urine, possibly because CYP3A4 is inhibited by GFJ (15). CYP3A4 enzyme protein and mRNA expression has been reported in ductal and seromucous/serous acinar cells in parotid, submandibular, and labial salivary glands (16). Future studies examining E, F, 6β-hydroxy-E, and 6β-hydroxy-F levels and their respective ratios in salivary effluents are warranted.

We hypothesized that intense muscular work would inhibit 11β-HSD2 activity and that this inhibition would be enhanced by GFJ intake. Our findings indicate that intense muscular work under CON inhibited 11β-HSD2 enzyme activity to the same extent as seen at baseline in the GFJ treatment group (Figure 2). On the other hand, intense muscular work under GFJ treatment did not inhibit enzyme activity beyond its corresponding baseline level. The decrease in the E:F ratio in the CON treatment could be due to decreases in physiological pH (11,12), and lactate has been reported to inhibit 11β-HSD2 activity in cultured intestinal cells (17). During intense muscular work, there is a transient perturbation in the acid-base balance that is related to lactic academia in which pH can drop below 7.0 (13), and this is associated with increased glycolytic flux and results in lactic acidemia with reciprocal and stoichiometric changes in bicarbonate. At exhaustion, our subjects reached a blood lactate level of ~11 mmol/L, a level that is probably not high enough to attenuate 11β-HSD2 enzyme activity. Alternatively, it is also plausible that 11β-HSD2 activity may be inhibited by substrate saturation at high or rapid increases in F (4-fold in the present study), as has been suggested for renal 11β-HSD2 activity in patients with ectopic Cushing’s disease (4,5) or as a result of a combination of both inhibitory mechanisms.

There is evidence suggesting a link between deficiencies in 11β-HSD2 activity and increased blood pressure in normotensive and hypertensive individuals (8,9), particularly in patients diagnosed with “apparent mineralcorticoid excess” (1,9). In our study, GFJ was associated with the inhibition of 11β-HSD2 enzyme activity but not with increased systolic or diastolic blood pressure compared to CON. Upon closer examination of the data, we found a moderate correlation between Sal-F (r = 0.658, *p* = 0.003) and diastolic blood pressure (DBP) and a slightly stronger correlation between Sal-F and ∆-DBP between the treatment groups (Figure 4A). The association between Sal-F and DBP agrees with the findings of other investigations (18). The significant inverse correlation (Figure 4B) between baseline E:F CON and ∆-E:F (baseline E:F CON – baseline E:F GFJ) suggests that individuals with the highest E:F ratio during the CON treatment were more sensitive to the inhibitory effects of GFJ. The present study was designed to examine the acute effects of GFJ and intense muscular exercise on the E:F ratios, not-the chronic effects of GFJ. Future studies should examine the effect of chronic GFJ ingestion on E:F ratios and blood pressure. While there is a case report linking high GFJ intake, the inhibition of 11β-HSD2 enzyme activity, and increases in high blood pressure (5), a randomized clinical trial suggested that chronic GFJ resulted in a borderline decrease in systolic blood pressure in overweight and obese men and women. That trial (19) did not report 11β-HSD2 enzyme activity surrogates, making it difficult to interpret their findings. Collectively, in the short-term, GFJ intake may decrease 11β-HSD2 enzyme activity and increase blood pressure in susceptible individuals, while sustained GFJ may have vasodilating effects that could negate the adverse effects of decreased 11β-HSD2 enzyme activity on blood pressure. Future studies should examine the long-term effects of regular grapefruit intake on 11β-HSD2 enzyme activity and blood pressure regulation in normotensive and hypertensive populations.

In conclusion, our findings suggest that: 1) short-term GFJ treatment inhibits 11β-HSD2 enzyme activity; 2) intense muscular work inhibits 11β-HSD2 enzyme activity under CON conditions to a similar extent as GFJ alone, and no additive/synergistic effect by intense muscular work and GFJ were noted; and 3) the associations between Sal-F and Sal-E levels and DBP during the GFJ trial should be interpreted cautiously and warrant further investigation.
